# Reducing Sexual Violence by Increasing the Supply of Toilets in Khayelitsha, South Africa: A Mathematical Model

**DOI:** 10.1371/journal.pone.0122244

**Published:** 2015-04-29

**Authors:** Gregg S. Gonsalves, Edward H. Kaplan, A. David Paltiel

**Affiliations:** 1 Yale School of Public Health, P.O. Box 208034, 60 College Street, New Haven, CT, 06520–8034, United States of America; 2 Yale School of Management, P.O. Box 208200, 135 Prospect Street, New Haven, CT, 06520, United States of America; University of Waterloo, CANADA

## Abstract

**Background:**

Sexual violence is a major public health issue, affecting 35% of women worldwide. Major risk factors for sexual assault include inadequate indoor sanitation and the need to travel to outdoor toilet facilities. We estimated how increasing the number of toilets in an urban township (Khayelitsha, South Africa) might reduce both economic costs and the incidence and social burden of sexual assault.

**Methods:**

We developed a mathematical model that links risk of sexual assault to the number of sanitation facilities and the time a woman must spend walking to a toilet. We defined a composite societal cost function, comprising both the burden of sexual assault and the costs of installing and maintaining public chemical toilets. By expressing total social costs as a function of the number of available toilets, we were able to identify an optimal (i.e., cost-minimizing) social investment in toilet facilities.

**Findings:**

There are currently an estimated 5600 toilets in Khayelitsha. This results in 635 sexual assaults and US$40 million in combined social costs each year. Increasing the number of toilets to 11300 would minimize total costs ($35 million) and reduce sexual assaults to 446. Higher toilet installation and maintenance costs would be more than offset by lower sexual assault costs. Probabilistic sensitivity analysis shows that the optimal number of toilets exceeds the original allocation of toilets in the township in over 80% of the 5000 iterations of the model.

**Interpretation:**

Improving access to sanitation facilities in urban settlements will simultaneously reduce the incidence of sexual assaults and overall cost to society. Since our analysis ignores the many additional health benefits of improving sanitation in resource-constrained urban areas (e.g., potential reductions in waterborne infectious diseases), the optimal number of toilets identified here should be interpreted as conservative.

## Introduction

Violence against women is a major global health issue [1, 2]. The World Health Organization (WHO) estimates that 35% of women worldwide have experienced physical and/or sexual violence. Approximately a third of all women who have been in a relationship have been physically or sexually assaulted by their intimate partners, and this accounts for the greatest proportion of sexual violence overall. However, 7% of women globally have been sexually assaulted by a non-partner. The highest rates of non-partner sexual violence are in industrialized countries and in Africa, with a lifetime prevalence of 12·6% and 11·9%, respectively [3]. Urban environments can often exacerbate risk for violence against women [4].

Prevention of violence against women requires a comprehensive response addressing multiple sources of risk [5]. This report addresses the relatively neglected consideration of inadequate access to nearby sanitation facilities. Recently, development and human rights organizations have pointed to inadequate local sanitation facilities as a key driver of women’s risk for physical or sexual assault [6, 7], where travel to and from toilets exposes them to waiting perpetrators of violence. According to the WHO and the United Nations Children’s Fund, as many as one in three women worldwide do not have access to safe toilet facilities [8].

We sought to estimate the costs of installing and operating additional sanitation facilities and the impact this might have on both the incidence and social burden of sexual assault. While we acknowledge the uncertainties associated with many of the parameters in our analysis, we have tried to address this through both one-way, deterministic as well as probabilistic sensitivity analyses (DSA and PSA). This study is the first quantitative analysis to look at the link between sexual violence and sanitation in terms of their impact on women and society and we hope it will spur other research to document the phenomenon in greater detail and to strengthen the evidence base for policy making.

## Materials and Methods

### Analytic overview

We developed a mathematical model that links the risk of a sexual assault to the number of available sanitation facilities and the total time a woman must spend walking to or from a toilet. We applied the model to Khayelitsha (an urban township of the City of Cape Town, South Africa), using publicly available data on population size and density, the epidemiology of sexual assault, and the geography and logistics of toilet usage. We used Khayelitsha as a case study because it is an example of an environment with high rates of sexual violence and inadequate sanitation facilities, because critical data elements are readily available, and because the health and safety issues associated with inadequate sanitation there have been the subject of extensive recent public debate [9]. Local, pedestrian transit is more common among women in urban townships when compared to their male counterparts [10]. While local, pedestrian transit to other facilities (e.g., stores, bus stops) may be associated with acts of violence against women, these trips almost always happen in daylight and when there is significant other foot traffic present in in the township. Such trips are inherently less risky. By contrast, trips to the toilet are less amenable to scheduling, subject to biological imperatives and happen at all times of day. They are, by their nature, solitary, private acts that often occur in remote locations. They have become particularly dangerous for women, as described in the literature cited above.

For purposes of this analysis, we defined a composite societal cost function, comprising both the burden of sexual assault and the costs of installing and maintaining public chemical toilets. We estimated this cost function using both published estimates of the social burden of sexual assault and publicly available information on the operating costs of toilet facilities. Taken as a whole, this framework permitted us to express total social costs as a function of the number of available toilets and to identify an optimal (i.e., a cost-minimizing) social investment in toilet facilities. Recognizing the uncertainty surrounding many of our input data assumptions, we conducted extensive sensitivity analysis, exploring the influence that alternative parameter values would have on the optimal number of toilets, the incidence of sexual assaults, and our estimate of total social costs.

### Modeling framework

A complete specification of the mathematical model and its application to Khayelitsha is provided in the Technical Appendix (S1 File). Briefly stated, exposure time and the incidence of sexual assault are assumed to be driven by the following factors: average demand for toilet usage (i.e., daily trips to the toilet); the number, location, and clustering of existing toilet facilities (which together determine the distance a woman has to walk to get to the nearest facility); average walking speed; and total reported assault rates (adjusted upwards to reflect the fact that only a fraction of all assaults are ever reported).

To estimate the magnitude of the total exposure time for all the women in a given district, we collected data from a number of sources: population and census figures from Statistics South Africa; assault data from the South African Police Service; toilet installation and annual operating costs from the City of Cape Town; and literature-based estimates of average walking speed and the economic cost of a sexual assault. Table 1 describes the model’s parameters, labeled with the symbols that correspond to the key equations described in the Technical Appendix (S1 File).

**Table 1:** Key parameters.

Parameter	Base Value	Source
Reported Annual Sexual Assaults	635	13
Estimated Percentage of Sexual Assaults Occurring Outdoors	30%	16
Estimated Percentage Sexual Assaults Reported	15%	14,15
Assumed Baseline Percentage of Outdoor Assaults Occurring En Route To/From Toilets	50%	
Proportion of Population Female	0.52	12
Estimated Total Assault Rate Per Exposure-Hour Walking To/From Toilets (*r*)	3.53 x 10^–5^	Appendix
Average Household Size	4	11
Average Number of Families Using a Single Toilet	17.5	21,22
Daily Toilet Visits per Woman	6	23
Average Human Walking Speed (kph)	5	17
Average Annual Cost of Chemical Toilet (in SA Rand) (*c*)	10315	22
Economic Cost of Sexual Assault (in US dollars)	240776 (A)	18
South Africa GDP Per Capita Based on Purchasing Power Parity (PPP in US dollars)	11028 (B)	19
United States GDP per capita based on (PPP in US dollars)	49965 (C)	19
Estimated Cost of Sexual Assault (in US dollars) (*s* = A x B / C)	53000	
Constant Linking Assaults To Number Of Toilets (*k*)	47485	Appendix

### Population and geography

Population data for Khayelitsha provided by Statistics South Africa were from the 2011 national census in GIS file format for 583 small area layers of the census. Small area layers (SAL) are an amalgamation of one or more enumeration areas (EA), which are the basic units for planning, executing and capturing census data. SALs were created to protect confidentiality in the dissemination of census data because EAs contain geo-coded references to individual dwellings. The average size of households and proportion of women in the township were based on studies commissioned by the City of Cape Town in 2006 and 2005 respectively [11, 12].

### Epidemiology of sexual assault

We constructed an estimate of the number of sexual assaults occurring en route to a toilet. Raw figures on the number of reported sexual assaults from 2003 to 2012 were obtained from data compiled by the South African Police Service (SAPS) stations at Khayelitsha, Harare and Lingelethu-West, all within the township [13]. We applied three adjustments to the SAPS data. First, we accounted for the under-reporting of sexual assault in South Africa, noting that the percentage of reported incidents is estimated to be approximately 15% of total cases [14, 15]. This estimate is derived from the 1998 South Africa Demographic and Health Survey (SADHS), which was a nationally representative sample of approximately 12,000 completed interviews with women between the ages of 15 and 49. The data from the 1998 SADHS documents provincial variations in reporting, with women in certain provinces, Mpumalanga, Northern Cape and Gauteng, more likely to report and, additionally, women with no education and African women less likely to report these crimes to police. This suggests that African women in Khayelitsha in the Western Cape, may underreport sexual assault, making the 15% figure an over-estimate of sexual assaults reported. Second, we restricted ourselves to sexual assaults that occur outdoors. We chose a value of 30% based on published estimates that assaults in open spaces, alleys and at public toilets themselves represent 27·2–44·5%, 2·2–6·2% and 1–3·8% of total assaults, respectively [16]. This estimate is based on surveillance data on 1401 rape cases collected from 1996–1998 at three medico-legal clinics in Gauteng province. While having more recent and site-specific information to craft both the estimates of reported incidents and those happening outdoors would be ideal, there is a pervasive lack of adequate epidemiological and surveillance data on sexual assault in South Africa, which makes this task difficult. Finally, we assumed that 50% of sexual assaults occurring outdoors could be attributed to periods of travel to and from sanitation facilities. Our assignment of 50% of these incidents to travel to and from toilets is arbitrary—there are no data on this parameter in the literature to allow an estimate to be established more rigorously—but we examined a wide range of alternative values in sensitivity analysis.

Total exposure time to toilet-related assault risk was obtained by dividing the model-deduced total travel distance for women based on the existing allocation of toilets by mean walking speed. While local estimates of mean walking speed were unavailable, recent studies have measured average human walking speeds at approximately five kilometers per hour [17]. A complete specification of our estimation procedure is provided in the Technical Appendix (S1 File). However, we note here that our approach requires some critical assumptions: first, that assaults occur while en route to or from—but not while at—the toilets; b) that exposure time is proportional to time spent walking; and c) that the rate of assaults/exposure-hour is a constant that remains unchanged by our efforts to reduce exposure time.

### Social costs of sexual assault

To our knowledge, there are no published assessments of the social costs of sexual assault anywhere in Southern Africa. While an extensive literature exists on the economic cost of crime in the United States and Europe, estimates of the cost of specific criminal offenses are limited and vary widely. This may reflect the breadth of differing methodologies that have been employed, including: unit costing, jury compensation, willingness-to-pay approaches, and benefit-cost analyses [18].

For our base case analysis, we assumed that the social cost per sexual assault in South Africa was US$53,000. This value was obtained from a recent, widely cited US study [18], which estimated both US$41,252 in tangible costs (e.g., medical expenses, lost earnings, legal adjudication, and corrections) and US$199,642 in intangible costs (e.g., pain and suffering, risk of homicide). We used the ratio of the South African and US per capita gross domestic products (22%) to adjust the US estimates downward, reflecting established methods for multinational comparisons of health and social values [19, 20]. Recognizing the large uncertainty in these estimates, we explored values ranging from $26,500 to $106,000 in sensitivity analysis.

### Location and cost of sanitation facilities

Sanitation facilities were assumed to be randomly distributed within each SAL. This random distribution was chosen for both practical and methodological reasons. First, there was no map of the location of these services in Khayelitsha to allow us to create a base case utilizing field data. Second, and more importantly, the shifting nature of urban environments makes a probabilistic assignment of toilets or other services more useful than deterministic ones. The baseline case assumed a ratio of toilet to persons at 1:70 based on a field study in a sub-district of Khayelitsha known as Monwabisi Park and a community social audit in Khayelitsha performed in 2013 [21, 22]. Toilets were assigned according to this ratio, in clusters of seven to each SAL based on its population. Typically, toilets in this setting are grouped in small clusters. We relied on personal observations of these informal arrangements to assume an average cluster size of seven toilets for our baseline analysis; however, we varied this value from 3·5 to 14 in sensitivity analysis. The average annual expenditure on an individual chemical toilet in South African rand is R10,315 (or US$ 1041·92 based on an exchange rate of 9·9 rand per dollar) and is based on data provided to the Social Justice Coalition by the City of Cape Town and documented in the social audit described above. We assumed an average of six round-trips to the toilets per day per woman [23].

### Sensitivity analysis

Because of our concerns about the reliability of our parameter estimates, we first conducted a one-way, deterministic sensitivity analysis (displayed as a tornado diagram) to identify the most influential parameters in our model, varying the parameters across credible ranges of uncertainty (Table 2). We then used the most influential parameters to conduct a probabilistic sensitivity analysis (PSA) using a Monte Carlo simulation with 5000 iterations [24]. Of particular note, we chose uniform distributions to reflect the magnitude of uncertainty about our estimates rather than utilizing ones (e.g., normal, beta, lognormal) that might imply greater knowledge of the shape of the distributions of these parameters. The parameters we considered in the PSA were: the number of trips to the toilet per day; the number of toilets in a cluster; the percentage of women in the township; walking speed; the economic cost of sexual assault; the percentage of sexual assaults reported; the percentage of sexual assaults occurring outdoors; the percentage of sexual assaults happening en route to or from sanitation facilities and; the cost of installation and maintenance of a toilet. We also used one-way sensitivity analysis to explore how additional investments in sanitation facilities would translate into both reductions in subsequent assaults and changes in the overall social costs.

**Table 2:** Parameter values in deterministic and probabilistic sensitivity analysis (SA).

Parameter	Base Value	Range in SA
Estimated Percentage of Sexual Assaults Occurring Outdoors	30%	20–40%
Estimated Percentage Sexual Assaults Reported	15%	5–25%
Assumed Baseline Percentage of Outdoor Assaults Occurring En Route To/From Toilets	50%	20–80%
Proportion of Population Female	0.52	0.50–0.54
Daily Toilet Visits per Woman	6	2–10
Average Human Walking Speed (kph)	5	3–7
Average Annual Cost of Chemical Toilet (in US dollars)	1042	521–1563
Estimated Cost of Sexual Assault (in US dollars)	53000	26500–79500
Cluster Size	7	1–13

## Results

All results obtained from mathematical calculations are rounded to reflect the precision of the original input data measurements. Currently, we estimate there are 5600 toilets in Khayelitsha, clustered together in roughly 800 separate locations (Table 3). The direct annual cost of operating these toilets is US$ 6 million. The average round-trip travel distance to and from a toilet is 210 meters; at six trips per day and 2·5 minutes per trip, this suggests an assault risk exposure time of 15 minutes per woman per day. This results in 635 sexual assaults per year, creating US$ 34 million in assault-related social costs. Thus, the combined annual social cost associated with the current allocation of toilets is US$ 40 million.

**Table 3:** Base case and optimal scenario results summary.

	Base Case	Optimal Scenario	% change
Number of Toilets	5600	11300	+100
Number of Sexual Assaults	635	446	-30
Mean Travel Distance To/From Toilets (meters)	210	147	-30
Toilet Installation and Maintenance Costs (US dollars millions)	6	12	+100
Social Cost of Sexual Assault (US dollars millions)	34	23	-30
Total Costs (US dollars millions)	40	35	-10

We estimate that costs could be minimized by increasing the number of toilets to 11300 in 1620 separate locations. Round-trip travel distance would be reduced to 147 meters, thus reducing total exposure time to 10 minutes per woman per day and lowering the number of sexual assaults to 446—a 30% decrease. While the direct cost of toilet installation and maintenance would rise to US$12 million, this would be more than offset by a decrease in the indirect social cost of sexual assault to US$ 23 million, for a total combined cost of $35 million, a 10% decrease in total costs from the current scenario.

**Fig 1:**
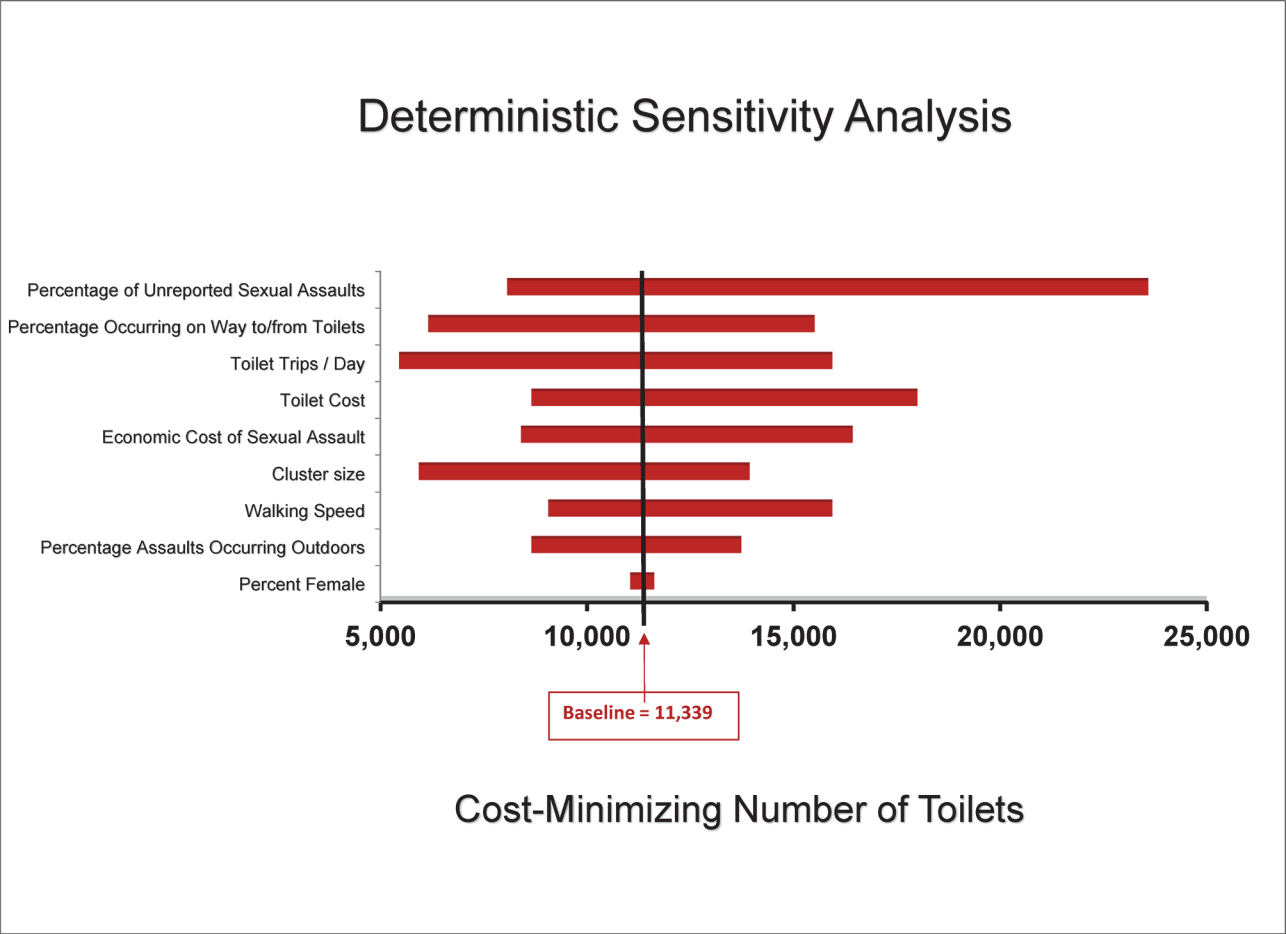
Deterministic sensitivity analysis (DSA).

The tornado diagram (Fig 1) summarizes the results of a series of one-way, deterministic sensitivity analyses. Each horizontal bar represents the range of “optimal toilets” produced by varying a given model parameter across its plausible range, as described in Table 2. The figure demonstrates that variation in the value of single parameters in our model still results in cost-minimizing numbers of toilets exceeding the baseline value of 5600. In the tornado diagram, the percentage of unreported sexual assaults, the number of round-trips to toilets per day and the percentage of sexual assaults happening en route to the toilets emerge as the three primary drivers of uncertainty in our model.

**Fig 2:**
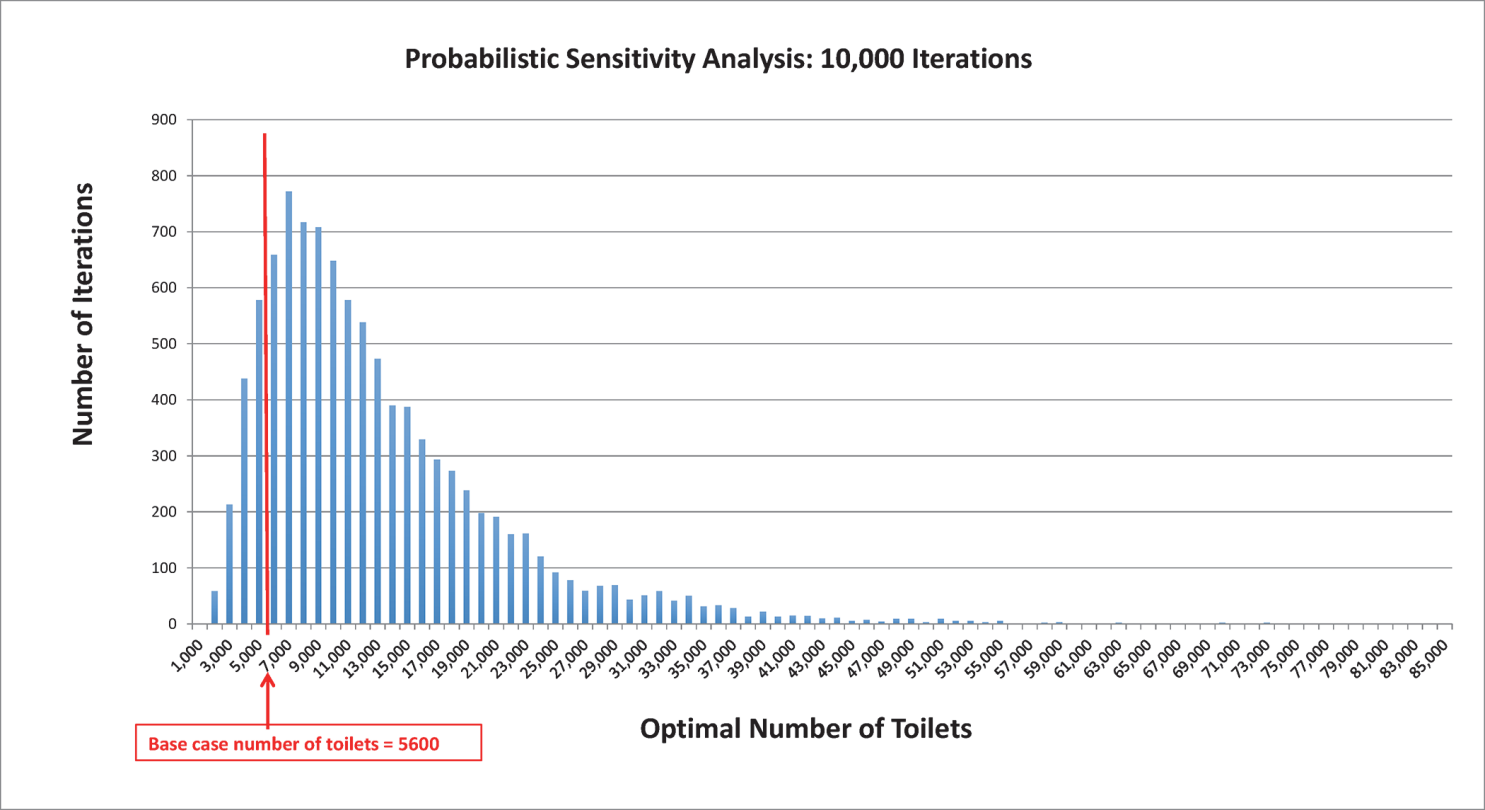
Probabilistic sensitivity analysis (PSA).

Using a probabilistic sensitivity analysis we assessed the magnitude of the uncertainty surrounding the point estimate for the cost-minimizing number of toilets. The PSA indicates that there is a high likelihood that many more toilets are required to minimize social cost than we currently estimate in the township. In over 80% of the 5000 simulations, the baseline number of 5600 toilets was exceeded (Fig 2).

**Fig 3:**
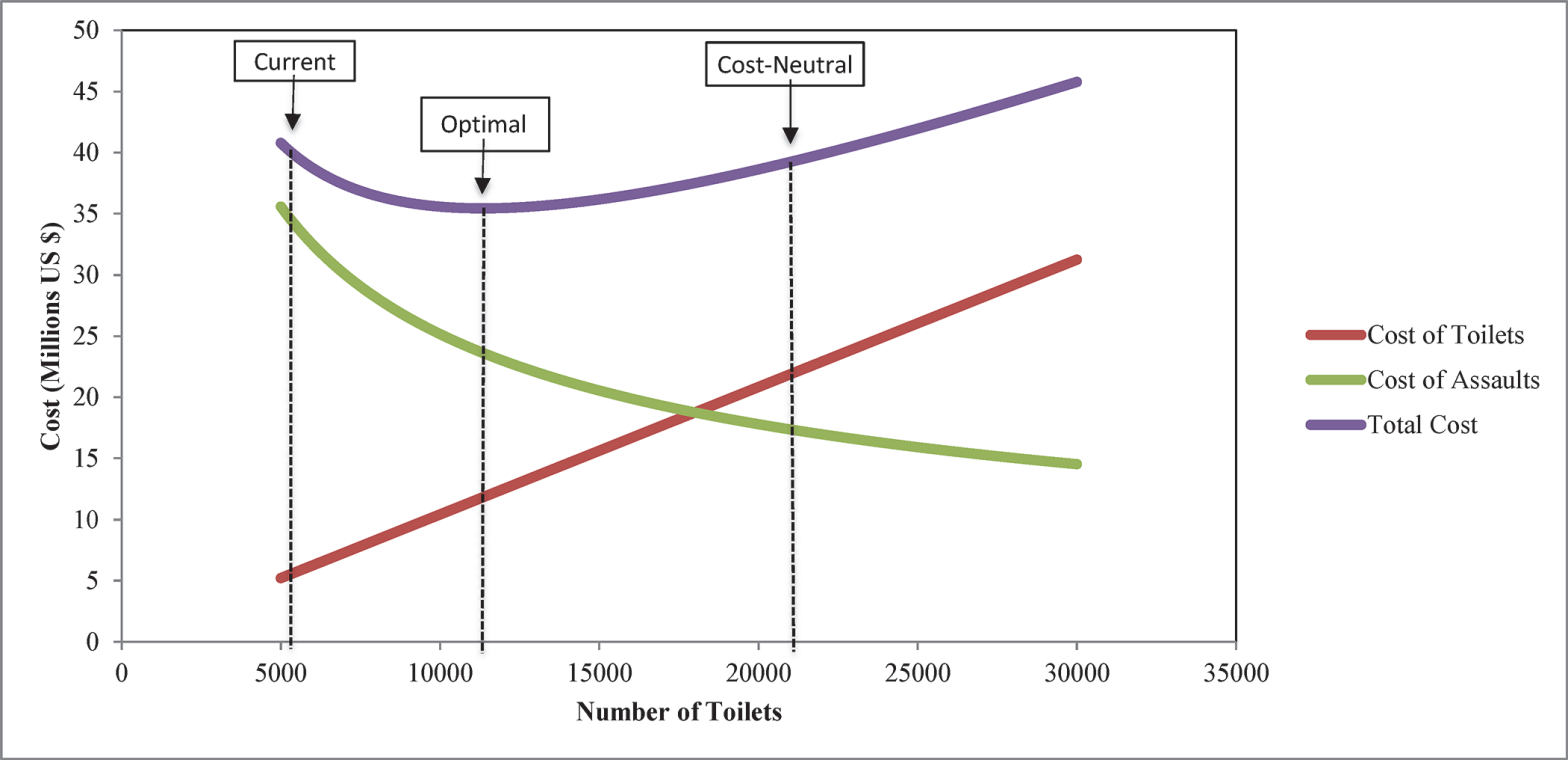
Toilet installation and maintenance costs and social cost of sexual assault (base case).

Finally, a simple, linear relationship exists between the total number of available toilets and the total cost of installing and maintaining those toilets (Fig 3). However, a more complicated relationship exists between the number of available toilets and the social costs of assault. This is because there are diminishing marginal benefits to increased investment in sanitation facilities; while doubling the number of facilities (from 5600 to 11200) produces 186 fewer assaults (from 635 to 449), tripling the number of toilets (to 16800) would only avert an additional 82 assaults. In addition, while overall social costs escalate at low and high number of toilets, the middle of the curve describing total social costs is relatively flat indicating a cost-neutral solution compared to the base case, in which up to 21400 toilets could be installed at no greater total social cost than associated with the original allocation and would decrease the number of assaults by nearly 50% to 325.

## Discussion

South Africa is rapidly urbanizing, as people in rural areas seek economic opportunity in cities. The United Nations estimates that more than 40% of the country’s population will reside in urban environments by 2050 [25]. However, ambivalence about rural-urban migration in South Africa has led to a lack of a coherent policy response to manage this demographic transition. In addition, the state has generally not supported urbanization directly as it has been wary of the short-term costs of providing additional services [26].

While the relationship between urbanization, poor sanitation, and infectious disease is well established [27], other important health effects are less well documented and explored. Urban areas pose special risks, particularly of gender-based violence towards women [4]. Our findings suggest that doubling of the number of toilets in Khayelitsha could produce dramatic reductions in both the number of rapes while, at the same time, reducing the overall costs to society. Our findings are all-the-more striking because our cost calculation does not take into account the many additional health benefits of improving sanitation in resource-constrained urban areas, particularly the potential reductions in morbidity and mortality associated with water-borne infectious diseases [28, 29]. The optimal number of toilets identified in this study should therefore be interpreted as a lower bound.

Sexual violence against women is only beginning to be recognized as a public health issue, whose epidemiology and economic burden need to be better described [5, 30]. Our findings should be interpreted with caution, in light of the large number of assumptions we made regarding the incidence, location, reporting, and social costs of sexual assaults in Khayelitsha. For example: we restricted our measure of exposure to time spent traveling to or from the toilets; we might also have considered time spent either waiting in line or currently using the facilities. We assumed that clustering has no effect on the direct cost of toilets, but has a strong effect on social cost, with larger clusters increasing exposure time and, by extension, the number of sexual assaults; the model does not reflect the potential benefits of clustering toilets and providing a degree of “safety in numbers.” Finally, we assumed the rate of sexual assault to be constant across all times of day.

It is a limitation of the study that so much empirical detail was missing. Much of this is due to the lack of attention these issues have received in the academic literature, particularly from a quantitative perspective. We conducted a thorough search to guide the development of our parameter estimates and in many instances data were simply lacking to make firmer approximations. The probabilistic sensitivity analysis we conducted relied on broad ranges for parameter values and used a uniform distribution for all those parameters we varied in the analysis. The probabilistic sensitivity analysis we conducted does not preclude the chance that the cost-minimizing number of toilets is at or below the baseline values we describe, but in the majority of the simulations, it was indeed the case that more toilets were required to minimize overall social cost.

In addition, because this is a model, we can only describe human behavior as it relates to the daily use of sanitation facilities in simple terms. In the real world, reaching the nearest toilet may require a circuitous route through the alleyways of the township; the nearest toilet may be in disrepair, and; individuals may visit a toilet as part of a longer trip to other destinations. All of these possible complications would, in fact, lengthen women’s exposure time and result in an even higher estimate of the optimal number of toilets. We also did not comprehensively describe the nature of women’s risk in urban settlements, where other locations such as shebeens (alcohol serving establishments) and the home are important loci of danger for women [15, 31]. We leave it to the reader to weigh the urgency of the epidemic of sexual violence, the robustness of our findings, and the value of the additional precision that might be achieved by delaying any policy intervention until better data can be obtained.

Two key behavioral assumptions deserve particular mention: First, we modeled sexual assaults as a constant rate per unit of exposure time. In doing so, we implicitly treated sexual violence as an opportunistic behavior that can be reduced by expanding the number of available toilets and thereby decreasing the number of exposure opportunities. If assault were more addictive in nature, then reducing the exposure time might induce assailants to modify their predatory behavior, driving them towards other locations, shifting the geography of women’s vulnerability and risk, and not affecting the overall number of sexual assaults. Studies from literature in the US suggest that predatory crimes, including sexual assault are tightly linked to place and opportunity and that interventions that are location-specific do not result in spatial displacement of the offenses, which supports our assumption here, though similar studies for South Africa are not available [32, 33]. Unless further research points to such behavior change, we believe that our simpler assumption of a constant assault rate is the more justifiable. For similar reasons, we also chose not to speculate on the possibility of endogeneity—i.e., that women avoid trips to the toilet because risk of sexual assault is high and that increasing the number of toilets might increase the number of trips—assuming instead that the number of toilet trips is physiologically determined. We recommend additional studies on the effects of access to sanitation on sexual violence and other public health priorities—including detailed analyses of women’s risk of sexual assault in these settings as well as the operational characteristics of service provision—to better inform future analyses, public health decision-making and urban planning.

Though our analysis was based on data from a single urban township in South Africa, the results are likely to be applicable to other informal settlements, both in South Africa and elsewhere in the developing world. In addition, these results may be useful in other settings where sexual violence is associated with women’s access to sanitation facilities, such as in refugee camps and temporary settlements established in the wake of a natural disaster [34, 35]. Our model incorporates location-specific data on the epidemiology of sexual violence; human population size, demographics and geography and; the cost of sanitation provision. These parameters could be changed to utilize data from other places to develop specific, additional local assessments. Though the social cost associated with deficiencies in sanitation provision in this study involves sexual violence alone, the model could be modified to include other social costs, including those associated with other violent and non-violent crime, infectious diseases, and other economic opportunity costs borne by residents of the township.

Sexual violence is endemic throughout the developed and developing world. Our model shows that improving access to sanitation facilities in urban informal settlements can simultaneously reduce both the number of sexual assaults and the overall cost to society.

## Supporting Information

S1 File Technical Appendix. (DOCX)

S2 File Gonsalves PSA PLOS ONE. (XLSX)

S3 File Gonsalves Primary Analysis PLOS ONE. (XLSX)
