# Supplementary material for: Reducing Sexual Violence by Increasing the Supply of Toilets in Khayelitsha, South Africa: A Mathematical Model
Source: PLoS One. 2015 Apr 29;10(4):e0122244. doi: 10.1371/journal.pone.0122244 (PMC4414450; doi:10.1371/journal.pone.0122244)
Supplement: S1 File — (DOCX) [file pone.0122244.s001.docx]

**Technical Appendix**

This appendix presents the mathematics underlying our model of the social costs of sexual assault and toilet provision. The model allows estimation of the expected number of sexual assaults that correspond to any particular allocation of toilets across different geographic zones, and further enables the determination of the number of toilets that minimizes the total costs of assaults and sanitary facilities. The appendix also details the empirical estimation of the parameters that were used to produce the results reported in the main text.

**Modeling Sexual Assaults in a Single Geographic Zone**

Sexual assault is a predatory behavior that can be characterized by an assault rate per unit of exposure time. In the context of this paper, women are exposed when traveling to and from toilets, while sexual predators prey on exposed women at a fixed rate. Total exposure time thus corresponds to the total time all women spend walking to and from toilets, and total assaults equal the product of total exposure time and the assault rate. Total exposure time depends upon the number of toilets: increasing (decreasing) the number of toilets will reduce (increase) the average travel distance to and from toilets; we assume a constant average walking speed; and higher (lower) assumed walking speeds serve to reduce (increase) total exposure time. With total assaults proportional to total exposure time via the assault rate, changing the number of toilets will change the number of assaults.

Letting *r* denote the rate of sexual assaults from all sexual predators per hour of exposure time, and *h*(*n*) be total exposure-hours per year over all women when there are *n* toilets in the geographic zone, annual assaults *A* can be expressed as

*A* = *r* × *h*(*n*) (1)

The dependence of annual assaults on the number of toilets thus reduces to determining how the annual number of exposure-hours depends upon the number of toilets in the zone.

Total exposure-hours can be broken down multiplicatively as

*h*(*n*) = # women × annual toilet visits/woman × 2*d*(*n*) / walking speed (2)

where *d*(*n*) is the average one-way distance from a random location to the nearest toilet in the zone. The number of women, annual toilet visits, and walking speed are all parameters directly estimated from data that do not depend upon the number of toilets. The distance per toilet visit depends upon the number of toilets and their configuration within the zone. The model presumes that clusters of toilets are distributed randomly within the geographic zone, and that travel between any two locations in the zone can be approximated by right-angle distance (which better approximates urban distances than straight-line travel) [1]. With these assumptions, it can be proven using geometrical probability arguments [2] that

(3)

where *g* is the number of toilets per cluster, and *a* is the spatial area of the geographic zone. Substituting equation (3) into equation (1) and (2) shows how increasing (decreasing) the number of toilets serves to decrease (increase) the annual number of assaults.

The derivation of *d(n_i_)* is based on a two-dimensional, homogeneous spatial Poisson process with right-angle distances. First we assume that toilets (really toilet clusters) are located within the zone in accord with a spatial Poisson process. Now consider a woman who resides at an arbitrary point in a zone. Since we approximate travel distance with the right-angle metric, we have a square of area *2r^2^* that encloses all points within *r* distance units of the woman’s residence. Within this area, the probability that there are exactly *k* toilets is given by:

The probability that the distance to the nearest toilet exceeds r is the same as the probability that there are no toilets within the above mentioned square, and thus the probability that the closest toilet is less than r distance units away, call this *F(r)*, is given by:

This is a Rayleigh probability distribution with parameter , which has mean

where is the average density of facilities, which in our case equals .

**Allocating Toilets Across Geographic Zones and Modeling Total Assaults**

Now consider an urban area that consists of *m* geographic zones with *n_i_* toilets distributed spatially at random in clusters of size *g* within zone *i* where the zone indicator *i* runs from 1 through *m*. The total number of toilets provided is denoted by *n*, and we presume that the toilets are allocated across zones in proportion to the population of each zone to the total urban population [3], that is,

*i* = 1, 2, …, *m* (4)

The average one-way travel distance from a random location to the nearest toilet in zone *i* follows from substituting the area of zone *i* (*a_i_*) and the number of toilets found in zone *i* (*n_i_*) into equation (3). Denote the resulting distance by *d(n_i_).* Then total exposure hours in zone *i*,*, h(n_i_)*, follows from equation (2) upon substituting *d(n_i_)* and the number of women residing in zone i into that equation. The average walking speed and annual number of toilet visits per woman are presumed equal across all zones. Annual assaults in zone *i*, which we denote as *A*(*n_i_*), are then given by equation (1) upon substitution of *h*(*n_i_*) where, lacking data to the contrary, we presume that the sexual assault rate per exposure-hour *r* is equivalent in all zones. The total number of assaults that result from the provision of *n* toilets across the entire urban area is then given by

(5)

Note that since the total number of toilets *n* appears as a square root factor in the denominator of *d*(*n_i_*) in every zone *i*, one can factor out all of the other terms in the formula for *A*(*n*) as a constant *k* to write

(6)

where

(7)

In conjunction with the parameter estimates discussed below, Equation (5) was employed to produce the figure in the main text.

**Modeling the Social Costs of Sexual Assault and Toilet Provision**

The annual cost of providing and maintaining a toilet is presumed to cost some amount *c*, thus the annual costs of providing *n* toilets just equal *c* x *n*. As detailed in the main text and below, we estimate the social cost of sexual assaults by adjusting published estimates of their direct and indirect costs in the United States downwards in accord with the ratio of South African to US per capita gross domestic product; denote the resulting social cost per sexual assault by *s*. The social cost of sexual assaults across the entire urban area when providing *n* toilets is then given by *s* $\times$ *A*(*n*). Consequently, the total social cost of sexual assault and toilet provision when supplying *n* toilets, *T*(*n*), is given by

*T*(*n*) = *c n* + *sA*(*n*). (8)

Equation (8) was used to produce the figure in the main text.

**Determining the Cost-Minimizing Number of Toilets**

Via equation (6) the total social costs of providing *n* toilets can be rewritten as

(9)

The cost-minimizing number of toilets *n*^*^ can be found by differentiating *T*(*n*) with respect to *n* and equating the result to zero. Doing so yields

(10)

The minimized total costs of sexual assault and toilet provision follow from inserting *n*^*^ into equation (9). Equations (9) and (10) produced our base-case result that the total costs of sexual assault and toilet provision could be minimized by increasing the number of toilets in Khayelitsha from 5596 to 11340, while these equations were also employed in the sensitivity analyses reported in Figs. 1 and 2 in the main text.

**Applying the Model to Khayelitsha: Data Sources and Parameter Estimation**

The model parameters were developed using data provided from Statistics South Africa and publicly available data retrieved through searches of Web of Science, Google Scholar, PubMed, reports from the City of Cape Town and the South African Police Service, as described in the main text of the paper.

**Population and geography**

Population data for Khayelitsha were provided by Statistics South Africa from the 2011 national census. The proportion of women in Khayelitsha (0.52) was obtained from figures developed by the City of Cape Town based on an earlier census in 2001 [4]. The average size of households in the township was based on a 2006 study commissioned by the City [5]. While this data may have also been obtained from the 2011 census, it was not offered to us by Statistics South Africa and we believe the estimates from the City of Cape Town for previous time periods were a reasonable substitute for our purposes. The number of women in an area was defined by multiplying the population of that area by the proportion of women in Khayelitsha as a whole. Data were provided in GIS file format for the 583 small area layers (SAL) of the census comprising the township (*m=583*); these SALs correspond to the “geographic zones” of the model derivation above. The area of each SAL was originally reported in decimal degrees and was converted into km^2^ using an Albers Equal Area Conic projection in ArcGIS 10.1.

**Epidemiology and economic assessment of sexual assault**

Information on sexual assault from 2003 to 2012 was obtained from data compiled by the South African Police Service (SAPS) stations at Khayelitsha, Harare and Lingelethu-West, all within the township[6]. Sexual assault is under-reported in South Africa and the percentage of reported incidents is thought to be approximately 15% of total cases [7, 8]. The percentage of sexual assaults happening outdoors—in open spaces, alleys or at public toilets themselves, ranges from 27.2-44.5%, 2.2-6.2% and 1-3.8%, respectively [9]. The estimated number of both reported and unreported sexual assaults estimated to occur outdoors en route to the toilets was constructed by averaging the annual number of sexual assaults from the SAPS data, then taking this figure as 15% of the total reported and unreported sexual assaults each year, of which 30% were assumed to occur outdoors. This figure was multiplied by 50%, so that half of the annual reported and unreported sexual assaults happening outdoors were assigned to periods of travel to and from sanitation facilities.

The sexual assault rate per hour (*r* in equation (1)) was calculated by dividing the number of both reported and unreported sexual assaults estimated to occur outdoors en route to the toilets by the total exposure time estimated from summing *h*(*n_i_*) over all SALs using equations (2) and (3) as applied to each SAL (that is, using the extant SAL-specific estimated number of toilets, area, and number of women). The daily number of one-way trips to the toilet was assumed constant and equal to 12 for all women. This figure was based on taking the mean value of the number of times women urinate (mean=5.6) and the mean value of the number of times women defecate (mean < 1) from studies in the literature, the former conducted in the United States and the later from the United Kingdom, since no local estimates were available [10, 11]. While local estimates of mean walking speed were unavailable, recent studies have measured average human walking speeds at approximately five kilometers per hour, which is the figure used for this study [12].

The economic cost of a single sexual assault was derived from estimates in the literature, which describe both tangible costs (e.g., medical expenses, lost earnings, legal adjudication, and corrections) and intangible costs (e.g., pain and suffering, risk of homicide) [13]. These estimates are based on data from the United States; no similar economic analyses are available for South Africa or elsewhere on the continent. For this analysis, the economic cost of a single sexual assault in Khayelitsha was discounted by a ratio of South African to United States per capita GDP of approximately 0.22 [14, 15].

**Sanitation facilities**

For the analysis, toilets were assumed to be randomly distributed within each SAL. The baseline case assumed a ratio of toilets to persons at 1:70 based on a field study in a sub-district of Khayelitsha known as Monwabisi Park and a community social audit in Khayelitsha carried out in 2013 [16, 17]. Within each SAL, toilets were assigned according to this ratio based on its population in clusters of seven (*g*=7). Typically, toilets in this setting are grouped in small clusters. We relied on personal observations of these informal arrangements to assume an average cluster size of seven toilets for our baseline analysis; however, we varied this value from 3·5 to 14 in sensitivity analysis. We are aware the number of toilets in Khayelitsha is not well documented, despite the social audits conducted to date. Anecdotal data suggests that the number could be as high as 74,000, well above the figure we use in the base case [18]. However, in our model, the sexual assault rate r is calculated, in part, from the extant number of toilets in each SAL, which become part of the constant k, in the formula for total costs. Thus, while an increase or a decrease in the base case number of toilets will affect the total costs in absolute terms, it would not affect the relative relationship between the base case and the cost-minimizing number of toilets; it would simply scale the optimal solution upwards or downwards.

The average annual expenditure on an individual chemical toilet (***a***) in South African rand is R10,315 or $1041 (1$=9.90605R) and is based on data provided to the Social Justice Coalition by the City of Cape Town and documented in the social audit described previously.

**References**

1. If xi and yi refer to the geographical coordinates of location i , then the right-angle distance between locations 1 and 2 equals as opposed to which computes straight line distance.

2. Larson RC, Odoni AR (2009) Urban operations research. 1981. Englewood Cliffs, NJ: Prentice-Hall.

3. In theory, a more efficient allocation of toilets to zones can be achieved by taking advantage of the different areas and hence population densities of the different zones, but there are no appreciable differences in the results obtained in Khayelitsha from simply assuming toilet allocation in proportion to the population, so we have retained this simpler approach.

4. QSJ Consultants (2006) Socio-economic profiling of urban renewal nodes: Khayelitsha and Mitchell’s Plain : executive summary. City of Cape Town, Information and Knowledge Management.

5. City of Cape Town (2005) A Population Profile of Khayelitsha: Socioeconomic Information from the 2001 Census. Cape Town, South Africa. Available: <https://www.capetown.gov.za/en/stats/CityReports/Documents/Population%20Profiles/A_Population_Profile_of_Khayelitsha_1052006142120_359.pdf>. Accessed 31 December 2014.

6. Institute for Security Studies (2012) SA Crime Stats. ISS|Crime Hub. Available: <http://www.issafrica.org/crimehub/stats>. Accessed 31 December 2014.

7. South Africa., Department of Health. (1999) South Africa Demographic and Health Survey 1998: preliminary report. [Pretoria]: Dept. of Health. Available from: <http://www.hst.org.za/sites/default/files/SADHS_1998_Full.pdf>. Accessed 31 December 2014.

8. Jewkes R, Abrahams N (2002) The epidemiology of rape and sexual coercion in South Africa: an overview. Social Science & Medicine 55: 1231–1244.

9. Swart L-A, Gilchrist A, Butchart A, Seedat M, Martin L (2000) Rape surveillance through district surgeon offices in Johannesburg, 1996-1998: Findings, evaluation and prevention implications. South African Journal of Psychology 30: 1 – 10.

10. Burgio KL, Engel BT, Locher JL (1991) Normative patterns of diurnal urination across 6 age decades. The Journal of urology 145: 728–731.

11. Heaton KW, Radvan J, Cripps H, Mountford RA, Braddon FE, et al. (1992) Defecation frequency and timing, and stool form in the general population: a prospective study. Gut 33: 818–824.

12. Browning RC, Baker EA, Herron JA, Kram R (2006) Effects of obesity and sex on the energetic cost and preferred speed of walking. Journal of Applied Physiology 100: 390–398.

13. McCollister KE, French MT, Fang H (2010) The cost of crime to society: New crime-specific estimates for policy and program evaluation. Drug and alcohol dependence 108: 98–109.

14. World Bank (2014) GNI, PPP (current international $). International Comparison Program database. Available: <http://data.worldbank.org/indicator/NY.GNP.MKTP.PP.CD>. Accessed 31 December 2014.

15. Commission on Macroeconomics and Health (2001) Macroeconomics and health: investing in health for economic development. Report of the Commission on Macroeconomics and Health: Executive Summary. World Health Organization.

16. Granfone M, Lizewski C, Olecki D (2008) Envisioning Endlovini: Water and Sanitation. WPI. Available: <http://wp.wpi.edu/capetown/files/2009/12/ATLAS-FINAL-PDF-Part-1-compressed-format.pdf>. Accessed 31 December 2014.

17. Social Justice Coalition (2013) Results of the Khayelitsha “Mshengu” Toilet Social Audit. Cape Town, South Africa. Available: <http://internationalbudget.org/wp-content/uploads/Social-Justice-Coalition-Report-of-the-Khayelitsha-Mshengu-Toilet-Social-Audit.pdf>. Accessed 31 December 2014.

18 Korte M (2014) Project to map Khayelitsha’s toilets could improve sanitation. GroundUp. Available: <http://groundup.org.za/article/toilet-mapping-khayelitsha-organisations-create-fault-monitoring-system_1962>. Accessed 31 December 2014.
